# Potential use of TG68 - A novel thyromimetic - for the treatment of non-alcoholic fatty liver (NAFLD)-associated hepatocarcinogenesis

**DOI:** 10.3389/fonc.2023.1127517

**Published:** 2023-02-23

**Authors:** Andrea Caddeo, Marina Serra, Francesca Sedda, Andrea Bacci, Clementina Manera, Simona Rapposelli, Amedeo Columbano, Andrea Perra, Marta Anna Kowalik

**Affiliations:** ^1^Department of Biomedical Sciences, University of Cagliari, Cagliari, Italy; ^2^Department of Pharmacy, University of Pisa, Pisa, Italy

**Keywords:** NAFLD, differentiation, liver preneoplastic lesions, thyroid hormone, thyromimetic, THRb agonist

## Abstract

**Introduction:**

Several lines of evidence suggest that the thyroid hormone signaling pathway is altered in patients with NAFLD and that pharmacological strategies to target the thyroid hormone/thyroid hormone nuclear receptor axis (TH/THR) in the liver may exert beneficial effects. In this study, we investigated the effect of TG68, a novel THRβ agonist, on rat hepatic fat accumulation and NAFLD-associated hepatocarcinogenesis.

**Methods:**

Male rats given a single dose of diethylnitrosamine (DEN) and fed a high fat diet (HFD) were co-treated with different doses of TG68. Systemic and hepatic metabolic parameters, immunohistochemistry and hepatic gene expression were determined to assess the effect of TG68 on THRβ activation.

**Results:**

Irrespectively of the dose, treatment with TG68 led to a significant reduction in liver weight, hepatic steatosis, circulating triglycerides, cholesterol and blood glucose. Importantly, a short exposure to TG68 caused regression of DEN-induced preneoplastic lesions associated with a differentiation program, as evidenced by a loss of neoplastic markers and reacquisition of markers of differentiated hepatocytes. Finally, while an equimolar dose of the THRβ agonist Resmetirom reduced hepatic fat accumulation, it did not exert any antitumorigenic effect.

**Discussion:**

The use of this novel thyromimetic represents a promising therapeutic strategy for the treatment of NAFLD-associated hepatocarcinogenesis.

## Introduction

Non-alcoholic fatty liver disease (NAFLD), the most common cause of chronic liver disease in Western countries (1), comprises a wide spectrum of clinical entities ranging from simple steatosis to non-alcoholic steatohepatitis (NASH), fibrosis, cirrhosis and, ultimately, to hepatocellular carcinoma (HCC) (2, 3). Recent meta-analyses revealed that the global prevalence of NAFLD is approximately 30% and it is increasingly growing worldwide (4). This unequivocally implicates that NAFLD is becoming the emerging risk factor for HCC (5). Unfortunately, patients with NAFLD-related HCC present more advanced tumor stage, lower eligibility for curative treatment, shorter survival time and higher rates of tumor recurrence (6, 7). Although several molecular mechanisms have been identified and pharmacological candidates are currently in advanced stages of clinical trials, there are still no approved pharmacological therapies for the treatment of NAFLD (8). This aspect seems even more critical as HCC is a cancer type with limited therapeutic options that confer only a modest improvement in overall survival (9). For these reasons, new therapies for NAFLD and NAFLD-related HCC are urgently needed. In this context, experimental and clinical studies suggested that alterations of the thyroid hormones (THs) signaling in the liver play a key role in the development and progression of NAFLD and HCC. THs, 3,5,3’-triiodo-L-thyronine (T3) and 3,5,3’,5’-tetraiodo-L-thyronine (thyroxine or T4), are essential regulatory molecules for normal growth, development and for maintaining metabolic homeostasis (10). Most of THs effects are mediated by nuclear receptors (THRs): thyroid hormone receptor α (THRα) and thyroid hormone receptor β (THRβ), whose distribution is heterogeneous among different tissues and/or during developmental stages (11). THRβ is the most abundant isoform in the liver where it mediates T3 effects on lipid metabolism and regulation of metabolic rate (12). As to NAFLD, several clinical investigations showed that overt and subclinical hypothyroidism and reduced THRβ expression correlated with NAFLD stage (13–15). Moreover, subclinical hypothyroidism and low-normal thyroid function were independent predictors of NASH and advanced fibrosis (16). Even if this correlation has been questioned by other studies that found a positive association of free T3 levels with the severity of hepatic steatosis and fibrosis (17, 18), exogenous T3 administration showed encouraging results in lowering hepatic fat content in various models of NAFLD in mice and rats (19, 20).

With regard to HCC, three independent case-control studies indicated that hypothyroidism represents a risk factor for human HCC (21–23). Moreover, severe local hypothyroidism was reported in rat hepatic preneoplastic lesions and in rat and human HCCs, suggesting that this condition may represent a favorable event for HCC development (24–26). The finding that exogenous T3 administration inhibits HCC progression (27) and induces an almost complete regression of advanced HCCs in rats (28), further strengthens the role of the TH/THR axis in hepatocarcinogenesis.

Nevertheless, these potentially therapeutic effects of T3 required to induce the anti-steatotic and anti-tumoral effects, occur whilst inducing a thyrotoxic state, including life-threatening tachyarrhythmias, muscle wasting, bone mass loss, all hampering the use of thyroid hormone. Since most of the harmful effects of T3, directed towards cardiovascular system, are mediated by THRα, THRβ-selective thyromimetics, such as GC-1 (Sobetirome), KB2115 (Eprotirome), the Hep-Direct prodrug VK2809 (MB07811) and Resmetirom (MGL-3196), which have reproduced T3-related biological effects on lipid metabolism without overt cardiotoxic effects, have been synthesized (20, 29–31). Recently, using GC-1 as a scaffold compound, our group reported the synthesis of a novel halogen free THRβ-selective agonist namely TG68 that showed a very high affinity for the THRβ (32). We demonstrated that TG68 strongly reduced hepatic fat accumulation *in vitro* (32) and *in vivo* in mice fed a high fat diet (HFD), in the absence of overt deleterious effects in extra-hepatic tissues, such as kidney or heart (33). On these premises, the aim of the present study was to investigate the effect of TG68 on an experimental model of NAFLD-associated hepatocarcinogenesis and to unveil the molecular mechanisms underlying the possible anti-tumorigenic effect of TG68.

## Materials and methods

### Rats and drug treatments

Four-week-old male Fischer-344 (F-344) rats were purchased from Charles River Italy (Calco, Italy). Rats were housed for two weeks at 22°C with free access to basal rodent diet (Mucedola s.r.l., Settimo Milanese, Italy) and drinking water, and with a 12-hours light/dark daily cycle before starting the experiments. All animal procedures were approved by the Italian Ministry of Health (the authorization codes are 1247/15-PR and 560/2019-PR), complied with national ethical guidelines for animal experimentation and were conducted in accordance with the guidelines of the local ethical committee for *in vivo* experimentation.

Three experimental protocols were adopted:

*Experimental Protocol 1.* Eleven rats given a single intraperitoneal dose of diethylnitrosamine (DEN, 150 mg/kg body weight) were fed *ad libitum* a high fat diet (HFD, 42% kcal/fat diet containing sucrose and 1,25% cholesterol, (Mucedola s.r.l.) for 30 weeks. HFD-fed rats were then split into two groups: group 1 (N=5) was maintained on HFD for further three weeks; group 2 (N=6) was fed a HFD plus TG68 (9.35 mg/kg in drinking water for 3 weeks). The dose 9.35 mg/kg was selected based on the dose-response to MGL-3196 on cholesterol lowering in DIO mice (30) and on our previous experiments (32).

*Experimental Protocol 2.* Twenty-three rats given a single dose of DEN as in Experimental Protocol 1 were fed *ad libitum* a HFD for 30 weeks and then split into three groups: group 1 (N=6) was maintained on HFD for further two weeks; group 2 (N=6) was fed a HFD plus TG68 (2.8 mg/kg, in drinking water) while a third group of rats (N=6) was fed a HFD plus TG68 (1.4 mg/kg in drinking water). Animals fed a basal diet were used as control group (N=5). Animals given TG68 were sacrificed 2 weeks later.

*Experimental Protocol 3*. Thirteen rats given DEN as in Experimental Protocol 1 and 2 were fed *ad libitum* a HFD for 39 weeks and then split into three groups: group 1 (N=4) was maintained on HFD for further two weeks; group 2 (N=5) was fed a HFD plus TG68 (2.8 mg/kg, in drinking water). A third group (N=4) was fed HFD plus Resmetirom (MGL-3196, 3 mg/kg in drinking water, MedChemExpress), for the last two weeks. All animals were sacrificed under isoflurane anaesthesia. Blood and tissues, including liver, heart and kidney, were collected.

### Analysis of serum triglycerides, cholesterol, glucose, aspartate aminotransferase, and alanine aminotransferase

Blood samples were collected from abdominal aorta. Serum was separated by centrifugation and tested for triglycerides (TGs), cholesterol (CH), glucose, aspartate aminotransferase (AST) and alanine aminotransferase (ALT) using a commercially available kit from Boehringer (Mannheim, Germany).

### Determination of hepatic TGs

Lipid extraction and measurement of TGs were performed according to Schroeder-Gloeckler et al. (34). Briefly, liver samples were homogenized in 8 volumes of deionized water and in 1 volume of 5 M NaCl. Subsequently, a 200 µl aliquot of the homogenate was mixed with 500 µl of methanol and 250 µl of chloroform. Following centrifugation, the organic phase was collected. Complete extraction of any residual lipids was achieved by re-extracting with 250 µl chloroform:methanol (9:1). The organic phase was separated by centrifugation and samples were dried at room temperature (RT). The lipids were dissolved in a solution of 90% isopropanol:10% Triton X-100 (2%) to disperse the TGs for assay. Hepatic TG content was measured colorimetrically using a kit from Sigma-Aldrich (TR0100; Sigma-Aldrich, Milan, Italy).

### Histology and immunohistochemistry

Immediately after sacrifice, liver, heart and kidney were weighted; sections were fixed in 10% buffered formalin and processed for histological analysis (hematoxylin and eosin, H&E) or immunohistochemistry (IHC). The remaining tissues were snap-frozen in prechilled 2-methylbutane in liquid nitrogen and stored at -80°C until use. To determine the hepatic neutral lipid content, frozen liver sections were stained with Oil Red O (ORO, Sigma Aldrich) for 15 min, rinsed with 60% isopropanol, and stained with Mayer hematoxylin. The ORO staining positive area for each sample was quantified by using ImageJ analysis software (National Institute of Mental Health, Bethesda, Maryland, USA). To investigate glucose-6-phosphatase (G6Pase) activity, 15 μm serial frozen sections were cut in a cryostat (Leica LMD6000), and stained for G6Pase and Glutathione S-transferase Placental form (GSTP) as previously described (27).

Paraffin-embedded tissues were cut into 4 μm sections, dewaxed, and hydrated. Slides were microwaved in citrate buffer pH 6.0 and incubated overnight with the primary antibodies: GSTP (#311, MBL International); Glucose-6-Phosphate Dehydrogenase (G6PD, ab87230, Abcam). Sections were incubated with the appropriate polymer DAKO Envision secondary antibody at RT. Signal was detected using the VECTOR^®^ NovaRED™ Peroxidase (HRP) Substrate Kit (Vector Laboratories). Sections were counterstained with Harris hematoxylin solution (Bio-Optica).

### Cytometric analysis

The area of GSTP-positive preneoplastic lesions was measured with ImageJ according to Abramoff et al. (35).

### RNA extraction and qRT-PCR

Total RNA was extracted from snap-frozen rat liver tissues by using Qiazol Lysis Reagent (Qiagen, Hilden, Germany) followed by RNeasy extraction kit (Qiagen). Extracted RNA was reverse-transcribed by using High-Capacity cDNA Reverse Transcription kit with RNase inhibitor (Thermo Fisher Scientific, Waltham, MA, USA). RNA was quantified by NanoDrop ND1000 (Thermo Fisher Scientific), while RNA integrity was assessed by Agilent Bioanalyzer 2100. Gene expression analysis was performed using TaqMan Gene expression Master Mix (Thermo Fisher Scientific) and the following specific TaqMan probes: *Dio1* Rn00572183_m1, *Myh*7 Rn00568328_m1, *Myh*6 Rn00568304_m1, *Cpt1a* Rn00580702_m1, *Acox1* Rn01460628_m1*, Pnpla2* Rn01479968_g1, *Fasn* Rn00569117_m1, *Dgat1* Rn00584870_m1, *Thrsp* Rn01511034_m1, *Klf9* Rn00589498_m1, *G6pd* Rn01529640_g1, *Gstp1* Rn00821792_g1, *Gapdh* 4351317. Each sample was run in triplicate and all measurements were normalized to *Gapdh*. Relative mRNA expression analysis for each gene was calculated by using the 2^-ΔΔCt^ method.

### Statistical analyses

All data were expressed as the mean ± standard deviation (SD). Differences between groups were compared by student’s t-test or by using one-way ANOVA followed by Tukey *post hoc* analysis using the GraphPad software (Prism 9) (La Jolla, California). P-values were considered significant at p < 0.05.

## Results

### TG68 caused a reduction of liver weight and steatosis

**Figure 1:**
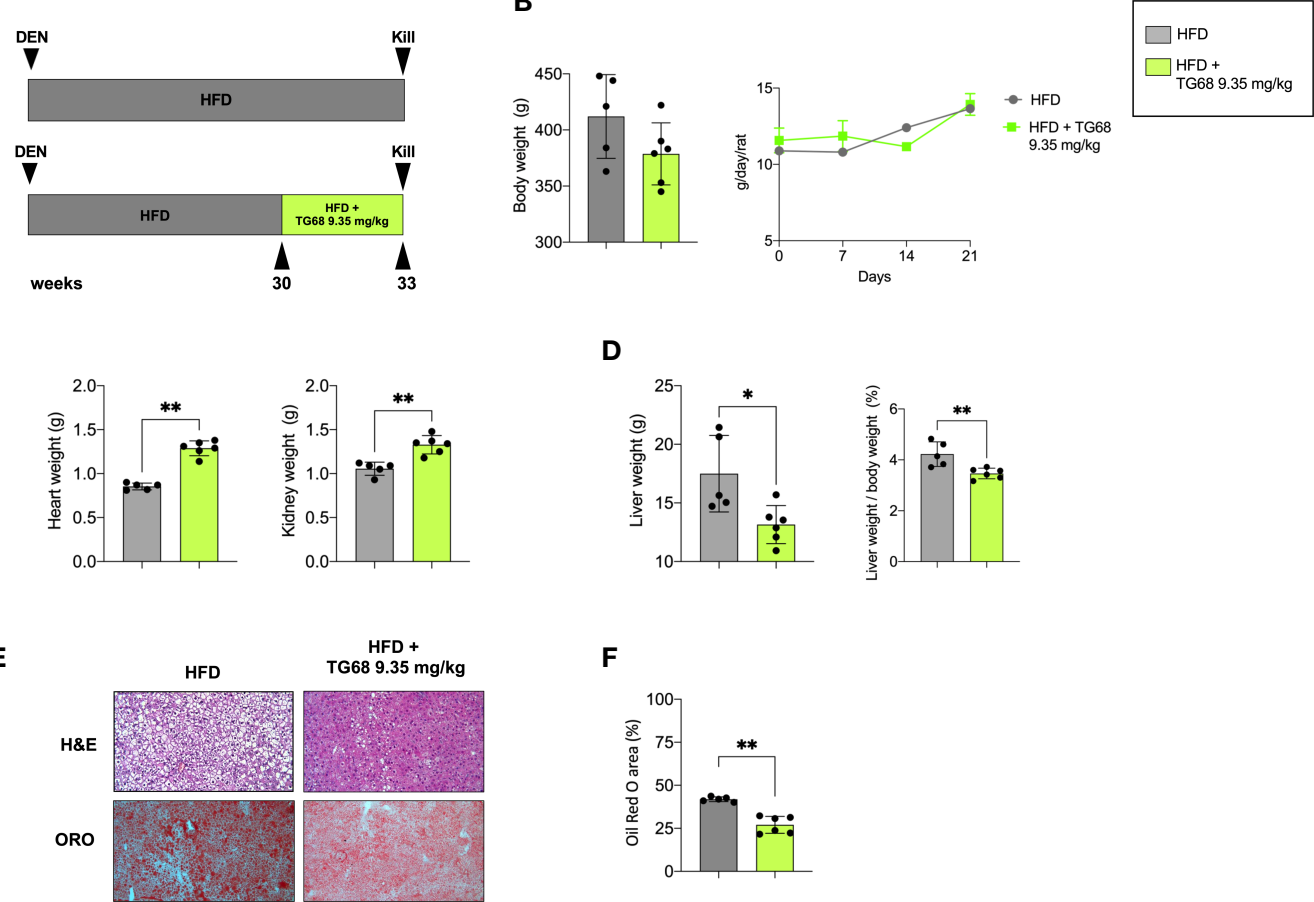
Effect of a three-week treatment with TG68 on kidney, heart and liver weight and hepatic steatosis. **(A)** Experimental design and timeline of the *in vivo* experiments; **(B)** Body weight and Daily food consumption throughout the whole experiment; **(C)** Heart weight and Kidney weight; **(D)** Liver weight and Liver weight/body weight ratio; **(E)** Representative images of liver sections stained with hematoxylin and eosin (H&E, 10x) or Oil Red O (ORO, 10x) at 9.35 mg/kg of TG68; **(F)** ORO staining positive area quantification by using ImageJ. Data were normalized to HFD alone. Groups were compared by student’s t-test. Values are shown as mean ± standard deviation of 5 to 6 rats/per group. *p<0.05, **p<0.01 HFD, High Fat Diet; DEN, Diethylnitrosamine; H&E, hematoxylin and eosin; ORO, Oil Red Staining.

To investigate the effect of TG68 on hepatic fat accumulation, in the first set of our experiments, we administered 9.35 mg/kg of the drug - dissolved in drinking water - to HFD-fed rats for the last 3 weeks (See experimental protocol in **Figure 1A**). TG68 caused a reduction of body weight, albeit not significant, compared to HFD alone, despite the fact that the food intake was similar in both the groups (**Figure 1B**). While an increase of heart and kidney weight was observed following TG68 treatment (**Figure 1C**), a significant reduction of liver weight and liver weight/body weight ratio compared to HFD-fed untreated rats was detected. (**Figure 1D**).

To investigate whether the observed reduction in liver weight was due to an amelioration of the hepatic steatosis, liver samples from both groups were subjected to comparative pathological analysis. The microscopic analysis of hematoxylin and eosin (H&E) stained sections confirmed the presence of steatosis in all the HFD livers from untreated mice (**Figure 1E**). ORO staining for neutral lipid content supported the histological observation highlighting the impressive reduction of hepatic fat content caused by TG68 (**Figures 1E, F**).

**Figure 2:**
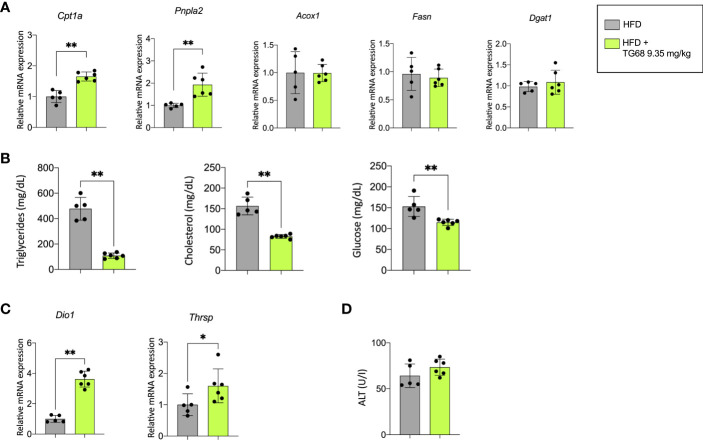
Effect of a three-week treatment with TG68 on lipid metabolism, serum triglycerides, cholesterol and glucose levels and mRNA levels of target genes of thyroid hormone receptor. **(A) ** Gene expression analysis of *Cpta1*, *Pnpla2*, *Acox1*, *Fasn, Dgat1* in rat livers exposed to Experimental Protocol described in Figure 1A. Gene expression is reported as fold-change relative to livers from rats fed HFD alone; **(B)** Effect of TG68 on serum triglycerides, cholesterol and glucose levels; **(C)** Gene expression analysis of *Dio1* and *Thrsp* in rat liver. Gene expression is reported as fold-change relative to livers from rats fed HFD alone; **(D)** Serum levels of Alanine aminotransferase (ALT). Groups were compared by student’s t-test. Values are shown as mean ± standard deviation of 5 to 6 rats/group. *p<0.05, **p<0.01. HFD, high fat diet; ALT, alanine aminotransferases.

The reduction of liver fat accumulation was accompanied by a significant up-regulation of *Phospholipase Domain Containing 2* (*Pnpla2)*, and *Carnitine Palmitoyltransferase-1* (*Cpt1a*), highlighting the effect of TG68 in decreasing the content of TGs on the one hand, and improving mitochondrial fatty acid oxidation in rats subjected to HFD on the other hand (**Figure 2A**). TG68 did not modify the mRNA levels of *Acyl-CoA oxidase1* (*Acox1)*, *Fatty acid synthase* (*Fasn*) and *Diacylglycerol O-acyltransferase1* (*Dgat1*) (**Figure 2A**). Taken together, these results suggested that TG68 increased lipolysis in steatotic liver without a major impact on lipogenesis. In addition to the effect on hepatic fat accumulation, TG68 caused a striking reduction of circulating TGs and CH accompanied by a significant reduction of blood glucose levels, compared with HFD rats (**Figure 2B**).

To verify whether the observed effects were associated with TG68-induced activation of THRs, the expression of *deiodinase* 1 (*Dio1*) and *thyroid hormone responsive* (*Thrsp*), two well-known THR target genes, were investigated. As shown in **Figure 2C**, the expression of *Dio1* and *Thrsp* was significantly increased following treatment with TG68.

Notably, unlike what was observed in the liver of HFD fed mice (33), microscopic analysis of rat liver did not exhibit any major sign of cell damage typically associated with NAFLD, such as cell swelling, Mallory-Denk bodies, acidophilic bodies or spotty necrosis. No detectable sign of liver cell injury following TG68 treatment was observed at light microscopic examination, as also shown by serum levels of ALT (**Figure 2D**). On the other hand, an increased cholangiocyte proliferation was occasionally observed in the liver of rats fed a HFD and co-treated with TG68.

### TG68 caused a reduction of the number and size of DEN-induced preneoplastic hepatic lesions

**Figure 3:**
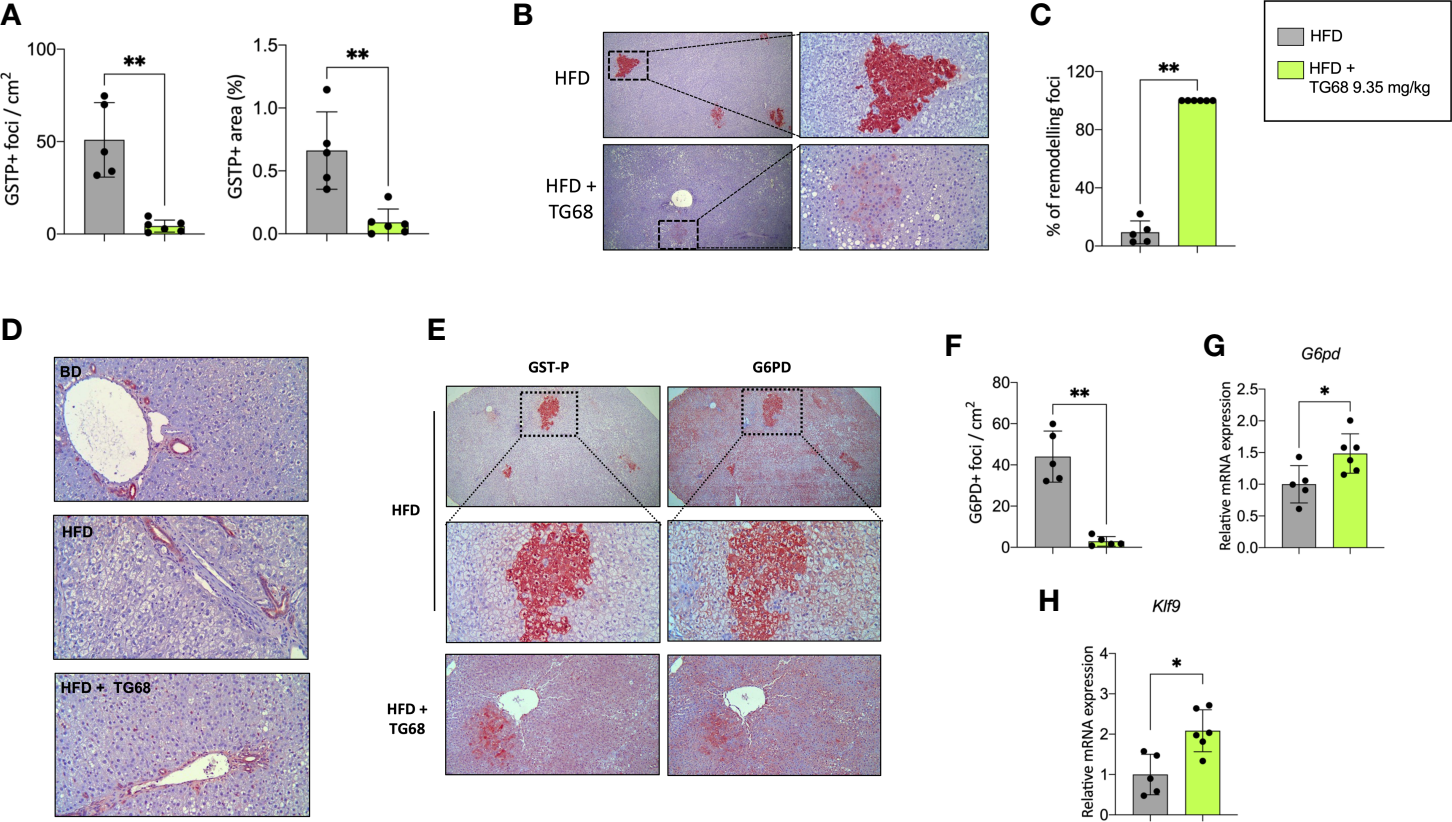
Effect of a three-week treatment with TG68 on DEN-induced preneoplastic foci. **(A)** Number of GSTP+-foci (left panel) and percentage of liver section occupied by GSTP+ lesions (right panel); **(B)** GSTP immunohistochemical staining of preneoplastic foci of rats fed HFD alone or co-treated with 9.35 mg/kg of TG68 (GSTP, 5X; Inset, 20X); **(C)** Percentage of remodeling foci in HFD and HFD+TG68 livers; **(D)** Representative pictures showing GSTP staining in hepatic bile ducts of rats fed Basal Diet (BD), HFD alone or co-treated with 9.35 mg/kg of TG68 (GSTP, 10x); **(E)** Immunohistochemistry on serial liver sections stained for GSTP and G6PD (GSTP/G6PD: 5x, 20x 10x); **(F)** Number of G6PD+-foci in rats fed HFD alone or co-treated with 9.35 mg/kg of TG68; **(G)** qPCR analysis of G6pd mRNA in rats fed HFD alone or co-treated with TG68; **(H)** qRT-PCR analysis of Klf9 mRNA levels in rats fed HFD alone or co-treated with TG68. Gene expression is reported as fold-change relative to livers from HFD-fed rats. The bar graphs represent mean values + SD of 5 to 6 rats/group. Groups were compared by using student’s t-test. *p<0.05, **p<0.01. HFD, high fat diet.

Experimental evidence has shown that T3 exerts an anti-tumorigenic effect at early and late stages of the process (27, 28). To investigate whether TG68 could exert a similar effect, we scored the presence of preneoplastic lesions immuno-stained against GSTP, the best marker for the identification of preneoplastic rat liver foci/nodules (36). As shown in **Figure 3A**, a single treatment with DEN followed by HFD feeding for 30 weeks resulted in a high number of GSTP+ foci (50.9/cm^2^), with 0.66% of the liver being positive for GSTP staining. Co-administration of TG68 for the last three weeks caused a significant reduction in the number (4.3/cm^2^, respectively) and the percentage of liver area occupied by GSTP-positive lesions (0.09%) (**Figure 3A**). Interestingly, while the vast majority of the GSTP-positive foci present in the liver of rats fed a HFD displayed an intense and homogeneous staining (persistent foci), almost all the ones observed in TG68-treated rats showed only a faint and discontinuous staining (remodeling foci) (**Figures 3B, C**), suggesting their reversion to a more differentiated phenotype following treatment with the thyromimetic.

The observed loss of GSTP staining caused by TG68 was not the consequence of a general transcriptional repression of GSTP expression by the drug, but a specific effect of TG68 on preneoplastic lesions. Indeed, immunohistochemistry showed no inhibitory effect of the drug on GSTP protein levels of bile ducts. (**Figure 3D**), thus indicating that the loss of GSTP immunostaining was due to the reacquisition of a differentiated phenotype of preneoplastic lesions. Further support to this proposition comes from the finding that TG68 caused an almost complete disappearance of preneoplastic lesions also when they were identified by G6PD immunostaining. Indeed, while almost all preneoplastic GSTP^+^ lesions in animals fed the HFD alone were also G6PD^+^ (**Figures 3E, F**), in rats co-treated with TG68 they were losing also G6PD positivity (**Figures 3E, F**), in spite of the increased hepatic mRNA levels of *G6pd* (**Figure 3G**). Taken together, these findings support the concept that TG68 caused the regression of preneoplastic foci by inducing a differentiated biochemical phenotype, and support the notion that activation of THRs by TG68 exerts an antitumoral effect. Support to the pro-differentiating effect of TG68 comes also from the finding of the enhanced expression of *Klf9 (* **Figure 3H**), a Kruppel-like factor that contains a thyroid hormone response element and is implicated in the regulation of the balance between pluripotency, self-renewal differentiation, and metabolism.

### Reduction of fat accumulation but not regression of preneoplastic lesions is achieved by further decreasing the dose of TG68

T3 and other thyromimetics have been shown to exert their effects on several organs, including heart and kidney (37). Indeed, as shown in **Figure 1C**, treatment with TG68 for three weeks caused a significant increase in both heart and kidney weight.

**Figure 4:**
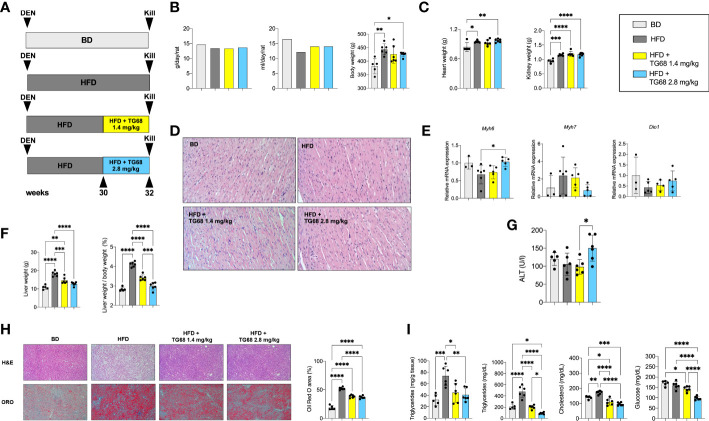
TG68 effectively reduces fat accumulation in the absence of significant cardiotoxic effects. **(A)** Experimental design and timeline of the *in vivo* experiments; **(B)** Daily food intake, water consumption and body weight of rats fed basal diet (BD), HFD alone or co-treated with 1.4 or 2.8 mg/kg of TG68; **(C)** Heart and kidney weight of rats treated as in A; **(D)** H&E staining of heart sections from rats treated as in A (H&E 20x); **(E)** qRT-PCR analysis of *Myh6*, *Myh7* and *Dio1 mRNA* in the heart of rats treated as in A; **(F)** Liver weight and liver weight/body weight ratio; **(G)** Serum ALT levels; **(H)** Representative images of liver sections stained with hematoxylin and eosin (H&E, 10x) or Oil Red O (ORO 10x) at 1.4 or 2.8 mg/kg of TG68, and quantification of ORO staining positive area by using ImageJ; **(I)** Hepatic content of triglycerides and serum levels of triglycerides, cholesterol and glucose. Gene expression is reported as fold-change relative to livers from rats fed a basal diet. The bar graphs represent mean value + SD of 3 to 6 rats/group. Groups were compared by using one-way ANOVA followed by Tukey *post hoc* analysis. *p<0.05, **p<0.01, ***p<0.001,****p<0.0001. BD, basal diet; DEN, Diethylnitrosamine; HFD, high fat diet.

Searching for experimental conditions that could avoid any possible impact of TG68 on extrahepatic organs, we adopted a protocol wherein rats given DEN and fed HFD were exposed to 1.4 or 2.8 mg/kg of TG68 for only 2 weeks (Experimental Protocol 2, **Figure 4A**). As shown in **Figure 4B**, no changes in food and water intake or body weight were detected at both the doses of the drug compared to HFD alone. Notably, heart and kidney weight were not affected compared to animals fed HFD alone (**Figure 4C**). Although no evidence of tissue damage could be observed by histological analysis (**Figure 4D**), to further investigate whether TG68 could cause damage to the heart through activation of THRs, we determined the mRNA levels of *Myosin heavy chain 6* (*Myh6*) and *Myosin heavy chain 7* (*Myh7*), two genes under TH control (38). As shown in **Figure 4E**, qRT-PCR analysis did not reveal any significant change in the cardiac expression of *Myh6* and *Myh7* in rats treated with both doses of TG68 compared to control rats. Notably, *Dio1* mRNA levels were not modified following TG68 administration (**Figure 4E**) suggesting a low delivery of the drug to cardiomyocytes, at least at the doses used in this experimental protocol.

On the other hand, both doses of TG68 caused a significant reduction of liver weight and liver weight/body ratio compared to HFD-fed animals (**Figure 4F**). These changes occurred in the absence of any significant sign of liver injury, as indicated by ALT serum levels as well as histologic analysis (**Figures 4G, H**). ORO staining confirmed that a massive reduction of fat accumulation accounted for the decreased liver weight observed with both the doses of TG68 (**Figure 4H**). Reduction of hepatic fat accumulation was accompanied by a significant decrease of the levels of TGs, CH and glucose in the blood (**Figure 4I**).

**Figure 5:**
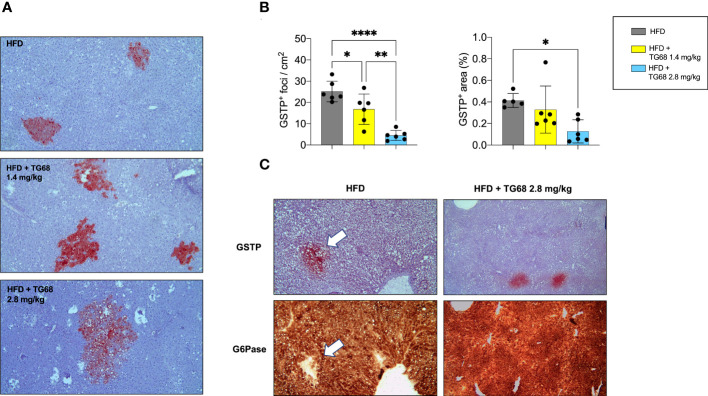
Reduction of fat accumulation does not always associate with regression of preneoplastic lesions. **(A)** Representative images of GSTP+ preneoplastic lesions from rats fed HFD alone or co-treated with 1.4 or 2.8 mg/kg of TG68 for the last 2 weeks (GSTP, 10x); **(B)** Number of GSTP+-foci per cm2 and percentage of liver occupied by GSTP+ lesions; **(C)** Representative image of serial liver sections. Arrows indicate that foci that are positive for GSTP are negative for G6Pase in rats fed HFD alone, but not in animals co-treated with TG68 (GSTP 10x; G6Pase 10x). The bar graphs represent mean values + SD of 5 to 6 rats/group. Groups were compared by using one-way ANOVA followed by Tukey post hoc analysis. *p<0.05, **p<0.01, ****p<0.0001. HFD, high fat diet.

Interestingly, while no clear difference on the regression of steatosis was observed between the two doses of TG68, a remarkably different effect on the regression of preneoplastic lesions was observed only with the dose of 2.8 mg/kg. Indeed, as shown in **Figures 5A, B** 2.8 mg/kg of TG68 led to a striking reduction of the number of GSTP+ foci and percentage of liver occupied by GSTP positive area (25/cm^2^ ± 4.8 and 0.4% in rats fed HFD alone *vs*. 4/cm^2^ ± 2.3 and 0.1% in rats HFD *vs*. TG68 co-treated animals). On the other hand, only a small decrease of the number of GSTP^+^ foci and no difference in the percentage of the liver occupied by GSTP^+^ lesions were observed in rats treated with the dose of 1.4 mg (17/cm^2^ ± 7.1 and 0.3%). Notably, while the intensely stained GSTP^+^ foci in the liver of rats fed HFD alone were virtually negative for G6Pase, an enzyme highly expressed by differentiated hepatocytes (**Figure 5C**), a faint staining of GSTP was observed in the preneoplastic lesions still present following TG68 treatment in concomitance with a re-expression of G6Pase (**Figure 5C**). This finding further supports the hypothesis that the anti-tumorigenic effect of TG68 is, at least in part, due to its ability to induce a switch of preneoplastic hepatocytes towards a differentiated phenotype.

### Reduction of fat accumulation but not regression of preneoplastic lesions are achieved by an equimolar dose of Resmetirom

**Figure 6:**
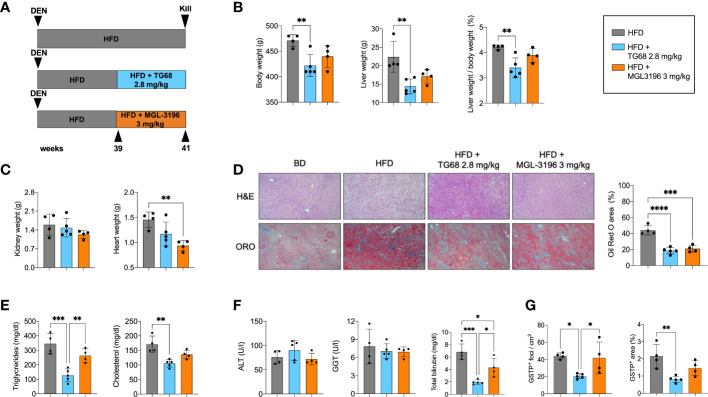
Resmetirom, unlike TG68, does not induce regression of preneoplastic foci. **(A)** Experimental design and timeline of the *in vivo* experiments; **(B)** Body weight, liver weight and liver weight/body weight ratio in rats fed HFD and then co-treated with 2.8 mg/kg of TG68 or 3.0 mg/kg of Resmetirom; **(C)** Heart and kidney weight of rats HFD alone or co-treated with 2.8 mg/kg of TG68 or 3.0 mg/kg of Resmetirom; **(D)** Representative images of liver sections stained with hematoxylin and eosin (H&E 10x) oor Oil Red O (ORO 10x) in rats treated as in A. Quantification of ORO staining positive area by using ImageJ; **(E)** Serum levels of triglycerides and cholesterol; **(F)** Serum levels of ALT, GGT and total bilirubin; **(G)** Number of GSTP^+^ foci and percentage of liver occupied by GSTP^+^ The bar graphs represent mean values + SD of 4 to 5 rats/group. Groups were compared by using one-way ANOVA followed by Tukey *post hoc* analysis. *p<0.05, **p<0.01, ***p<0.001,****p<0.0001. ALT, alanine aminotransferase; DEN, Diethylnitrosamine; HFD, high fat diet; MGL-3196, Resmetirom; GGT, gamma-glutamyl transpeptidase.

Next, we investigated whether i) TG68 could exert its anti-tumorigenic effect also at later stages of the carcinogenic process, and ii) a similar effect could be exerted also by Resmetirom (MGL-3196), a THRβ agonist that provided significant reductions in liver fat, low-density lipoprotein cholesterol and other atherogenic lipids *vs*. placebo in a Phase II trial (30). As illustrated in the Experimental Protocol 3 (**Figure 6A**), the rats exposed to DEN and fed a HFD for 39 weeks were then given either TG68 (2.8 mg/kg) or MGL-3196 (3 mg/kg) for 2 weeks. As shown in **Figure 6B**, only TG68 caused a decrease of body weight as well as of liver weight and liver weight/body weight ratio. Notably, a slight decrease of heart and kidney weight was observed with TG68 that was more pronounced with MGL-3196, compared to HFD-fed rats (**Figure 6C**). Both drugs strongly reduced the lipid content in the liver as detected by histological analysis and ORO staining (**Figure 6D**). Furthermore, TG68 also led to a decline in the levels of TGs and CH, although only the treatment with TG68 caused a statistically significant reduction of these parameters compared to the HFD group (**Figure 6E**). On the other hand, while both the drugs did not modify the serum levels of ALT or GGT, they caused a strong reduction of total bilirubin (**Figure 6F**).

Subsequently, we investigated and compared the effect of TG68 and MGL-3196 on DEN-induced preneoplastic foci. As shown in **Figure 6G**, at 41 weeks after DEN treatment the number of GSTP^+^ foci and the percentage of area occupied by GSTP^+^ foci in rats treated with HFD alone were much higher than those observed at 33 weeks (43 *vs*. 25 and 2.1 *vs*. 0.6, respectively). Interestingly, TG68 exerted a striking anti-tumorigenic effect even at this later stage of hepatocarcinogenesis. Indeed, it caused a 50% decrease in the number of GSTP^+^ foci (23/cm^2^ *vs*. 44/cm^2^) and an even stronger reduction in the % area occupied by preneoplastic lesions (0.8% *vs*. 2.1%). On the opposite, no significant change in the number of GSTP^+^ foci (42/cm^2^) or in the % area occupied by these lesions (1.5%) was induced by MGL-3196.

## Discussion

In the present study, we investigated the effect of a novel halogen free THRβ-selective agonist TG68 on hepatic steatosis and regression of preneoplastic lesions in rats exposed to DEN and HFD. In the last years, growing evidence has demonstrated that thyroid hormones and THRs are implicated in human HCC development and progression (21–23), and that severe local hypothyroidism takes place in rat hepatic pre- and neoplastic lesions, as well as in human HCCs, suggesting that this condition may represent a favorable event for HCC development (26–28). As to NAFLD, an emerging risk factor for HCC, the incidence of hypothyroidism resulted higher in patients with NAFLD/NASH compared to age-matched controls (15, 39). In this context, previous reports in animal models demonstrated that T3 exerts an anti-tumorigenic effect at early and late stages of hepatocarcinogenesis associated with a switch from Warburg to oxidative metabolism and loss of markers of poorly differentiated hepatocytes (27, 28). Moreover, T3 suppressed HCC onset in DEN-treated mice *via* activation of autophagy (40) and in HBV-encoded X protein-induced hepatocarcinogenesis (41). With regard to THRβ agonists, it has been observed that the treatment with GC-1 strongly reduced the number of preneoplastic foci generated in two different experimental models of liver carcinogenesis, the Resistant-Hepatocyte model and a nutritional model consisting in the feeding the choline-methionine deficient diet (42). In the current study, we applied an experimental protocol consisting in the administration of the initiating agent DEN and feeding a HFD diet. As already reported, chronic exposure of animals to a HFD closely recapitulates the complex pathological events associated with NAFLD in humans (43).

Here, we report that a short-term (two/three weeks) treatment of rats fed a HFD with a liver-selective THR-β agonist TG68 not only led to a reduction of hepatic fat accumulation and of serum triglycerides and cholesterol, but also caused a significant reduction of the number and size of DEN-induced preneoplastic hepatic lesions. We demonstrated that TG68 negatively influenced the carcinogenic process through an induction of a differentiation program of preneoplastic hepatocytes, as indicated by the loss of the preneoplastic marker GSTP, which is absent in differentiated hepatocytes.

The TG68-induced shift towards a differentiated phenotype was further supported by histochemical analysis showing reacquisition of G6Pase activity, an established marker of differentiated hepatocytes. Furthermore, the regression of preneoplastic lesions was associated with a return to a differentiated phenotype and was also sustained by the enhanced expression of Klf9, a transcription factor involved in the regulation of the balance between pluripotency, self-renewal and differentiation (44). As reported by Cvoro et al. (45), THRs cooperate with Klf9 to regulate hepatocyte differentiation and THR activation leads to KLF9 induction in transformed and non-transformed liver cells, and in stem cells. We also report that TG68 caused the regression of preneoplastic lesions by inducing a differentiated biochemical phenotype, as we observed an almost complete disappearance of G6PD, the rate-limiting enzyme of the oxidative branch of the pentose phosphate pathway (PPP). Remarkably, in our previous study we showed that an increase in G6PD expression in HCC patients was significantly associated with high-grade HCCs, and positively correlated with metastasis formation and decreased overall survival (46).

Although previous studies have already demonstrated the effectiveness of several THRβ agonists in reducing hepatic steatosis in animal models (47–49), the effect of THRβ-selective thyromimetics on the regression of preneoplastic lesions remained largely unexplored. The importance of the results obtained in our study regards the fact that at present there are no FDA-approved drugs for the treatment of NAFLD and only limited therapeutic options are currently available for this tumor type.

In our study, we investigated also whether reactivation of the T3/THR axis in the preneoplastic lesions by another THRβ agonist – Resmetirom (MGL-3196) - may exert the same effect on the regression of preneoplastic lesions induced by the DEN+HFD regimen. Resmetirom, a liver-directed THRβ agonist orally administered, entered a Phase 3 clinical trial (8). In a recent study, Resmetirom-treated patients showed a significant reduction of hepatic fat compared with placebo (30). Moreover, treatment with MGL-3196 reduced markers of fibrosis in adults with biopsy-confirmed NASH (50). In our previous study (33), MGL-3196 and TG68 shared the capacity to reduce hepatic steatosis in mice fed a HFD diet. In the current study, while both drugs strongly reduced the content of fat accumulation in the rat liver, only TG68 exerted a striking anti-tumorigenic effect, as no significant change in the number of GSTP+ foci was induced by MGL-3196. Further studies aimed at the elucidation of this different effect on the regression of preneoplastic lesions between MGL-3196 and TG68 are needed.

While considering the potential therapeutic use of THRβ-selective thyromimetics for NAFLD and NAFLD-related HCC, adverse effects on the heart should be considered. In this regard, a relevant observation stemming from this study is the lack of toxicity of TG68 on extra-hepatic organs, such as the heart. Indeed, while one of the most important adverse effects limiting the clinical use of thyroid hormone is its cardiotoxicity, neither macroscopic nor histological analyses of the cardiac tissue showed detectable signs of toxicity after treatment with this THRβ-selective thyromimetic. Based on these observations and on the finding that no change of the expression of *Myh6*, a target of activated THRs (38) occurred following TG68, we conclude that TG68 is sufficiently safe for use in long-term therapies. Nevertheless, other preclinical studies are required prior to its use in clinical trials.

**Figure 7:**
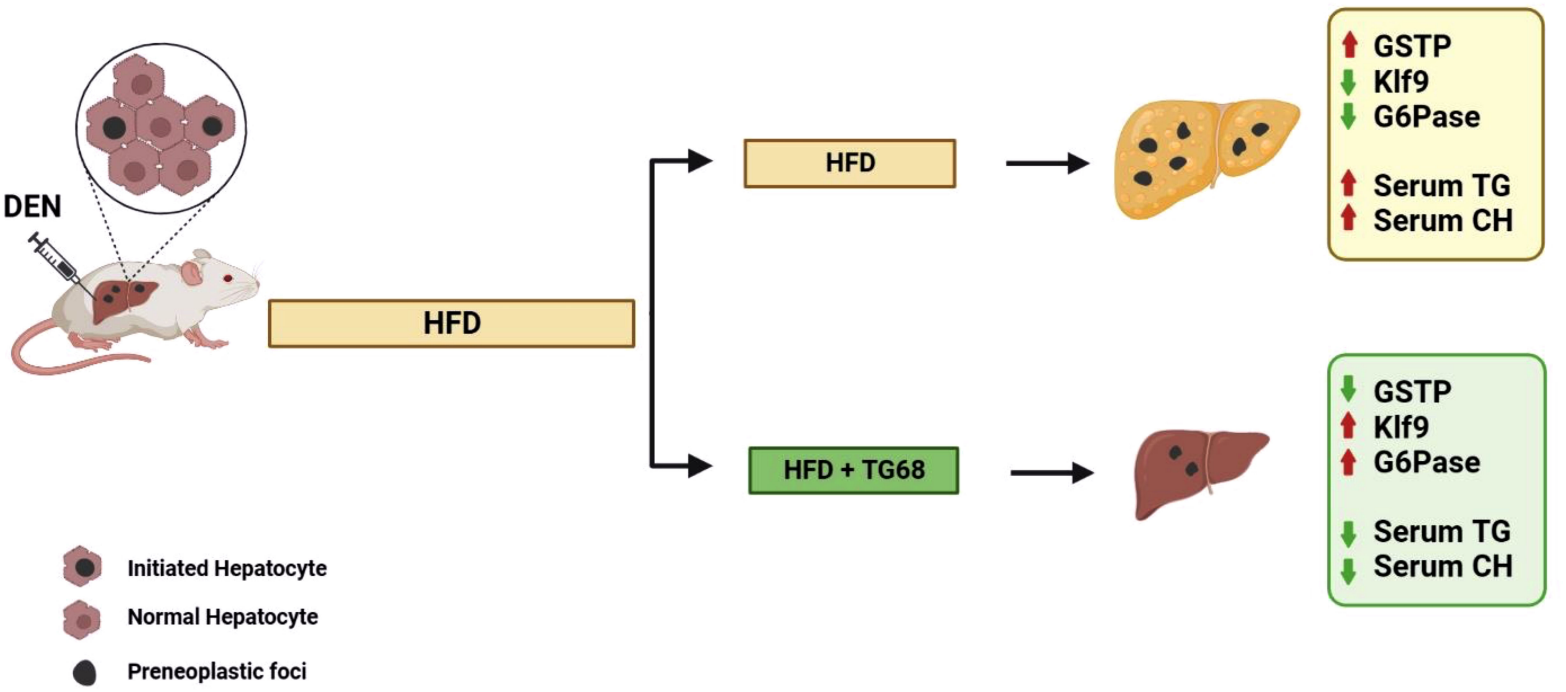
A schematic representation of the effect of TG68 on hepatic steatosis and DEN-induced hepatocarcinogenesis. Treatment with TG68 led to a significant reduction in hepatic steatosis, circulating triglycerides, cholesterol and caused regression of DEN-induced preneoplastic lesions associated with a differentiation program. DEN, Diethylnitrosamine; HFD, High Fat Diet; GSTP, placental form of glutathione-S-transferase; KLF9, Kruppel-like factor 9; G6Pase, Glucose-6-phosphatase; TGs, Triglycerides; CH, Cholesterol. Figure was created with BioRender.com.

In the light of the lack of approved pharmacological strategies for NAFLD and limited therapeutic options for NAFLD-related HCC, the results obtained in the present study suggest that the novel liver THRβ agonist TG68 might represent an attractive candidate for the treatment of NAFLD and NAFLD-related HCC (**Figure 7**).

## Data availability statement

The raw data supporting the conclusions of this article will be made available by the authors, without undue reservation.

## Ethics statement

All animal procedures were approved by the Italian Ministry of Health (the authorization codes are 1247/15-PR and 560/2019-PR), complied with national ethical guidelines for animal experimentation and were conducted in accordance with the guidelines of the local ethical committee for *in vivo* experimentation.

## Author contributions

ACa, AP, MK performed the *in vivo* experiments and analyzed data. AP performed histopathologic classification. ACa, MS analyzed gene expression profile. ACa, MK, MS, FS performed histochemistry and immunohistochemistry. AB, SR synthesized TG68 for *in vivo* studies. CM proofread the manuscript. ACo, AP, MK conceived and supervised the study, provided funding, wrote the manuscript. All authors contributed to the article and approved the submitted version.

## References

[1] Asrani SK Devarbhavi H Eaton J Kamath PS. Burden of liver diseases in the world. J Hepatol (2019) 70(1):151–71. doi: 10.1016/j.jhep.2018.09.014 30266282

[2] Ludwig J Viggiano TR McGill DB Oh BJ. Nonalcoholic steatohepatitis: Mayo clinic experiences with a hitherto unnamed disease. Mayo Clin Proc (1980) 55(7):434–8. 7382552

[3] Michelotti GA Machado MV Diehl AM. NAFLD. NASH and liver cancer. Nat Rev Gastroenterol Hepatol (2013) 10(11):656–65. doi: 10.1038/nrgastro.2013.183 24080776

[4] Younossi ZM Koenig AB Abdelatif D Fazel Y Henry L Wymer M. Global epidemiology of nonalcoholic fatty liver disease-meta-analytic assessment of prevalence, incidence, and outcomes. Hepatology (2016) 64(1):73–84. doi: 10.1002/hep.28431 26707365

[5] Shah PA Patil R Harrison SA. NAFLD-related hepatocellular carcinoma: The growing challenge. Hepatology (2023) 1;77(1):323–38. doi: 10.1002/hep.32542 PMC9970023 35478412

[6] Younossi ZM Otgonsuren M Henry L Venkatesan C Mishra A Erario M. Association of nonalcoholic fatty liver disease (NAFLD) with hepatocellular carcinoma (HCC) in the united states from 2004 to 2009. Hepatology (2015) 62(6):1723–30. doi: 10.1002/hep.28123 26274335

[7] Mohamad B Shah V Onyshchenko M Elshamy M Aucejo F Lopez R. Characterization of hepatocellular carcinoma (HCC) in non-alcoholic fatty liver disease (NAFLD) patients without cirrhosis. Hepatol Int (2016) 10(4):632–9. doi: 10.1007/s12072-015-9679-0 26558795

[8] Xu X Poulsen KL Wu L Liu S Miyata T Song Q. Targeted therapeutics and novel signaling pathways in non-alcohol-associated fatty liver/steatohepatitis (NAFL/NASH). Signal Transduct Target Ther (2022) 7(1):287. doi: 10.1038/s41392-022-01119-3 35963848 PMC9376100

[9] Huang A Yang XR Chung WY Dennison AR Zhou J. Targeted therapy for hepatocellular carcinoma. Signal Transduct Target Ther (2020) 5(1):146. doi: 10.1038/s41392-020-00264-x 32782275 PMC7419547

[10] Yen PM. Physiological and molecular basis of thyroid hormone action. Physiol Rev (2001) 81(3):1097–142. doi: 10.1152/physrev.2001.81.3.1097 11427693

[11] Lazar MA. Thyroid hormone receptors: Multiple forms, multiple possibilities. Endocr Rev (1993) 14(2):184–93. doi: 10.1210/edrv-14-2-184 8325251

[12] Forrest D Vennström B. Functions of thyroid hormone receptors in mice. Thyroid (2000) 10(1):41–52. doi: 10.1089/thy.2000.10.41 10691312

[13] Chung GE Kim D Kim W Yim JY Park MJ Kim YJ. Non-alcoholic fatty liver disease across the spectrum of hypothyroidism. J Hepatol (2012) 57(1):150–6. doi: 10.1016/j.jhep.2012.02.027 22425701

[14] Ludwig U Holzner D Denzer C Greinert A Haenle MM Oeztuerk S. Subclinical and clinical hypothyroidism and non-alcoholic fatty liver disease: A cross-sectional study of a random population sample aged 18 to 65 years. BMC Endocr Disord (2015) 15:41. doi: 10.1186/s12902-015-0030-5 26276551 PMC4536732

[15] Krause C Grohs M El Gammal AT Wolter S Lehnert H Mann O. Reduced expression of thyroid hormone receptor β in human nonalcoholic steatohepatitis. Endocr Connect (2018) 7(12):1448–56. doi: 10.1530/EC-18-0499 PMC6300861 30496129

[16] Kim D Kim W Joo SK Bae JM Kim JH Ahmed A. Subclinical hypothyroidism and low-normal thyroid function are associated with nonalcoholic steatohepatitis and fibrosis. Clin Gastroenterol Hepatol (2018) 16(1):123–131.e1. doi: 10.1016/j.cgh.2017.08.014 28823829

[17] van den Berg EH van Tienhoven-Wind LJN Amini M Schreuder TCMA Faber KN Blokzijl H. Higher free triiodothyronine is associated with non-alcoholic fatty liver disease in euthyroid subjects: The lifelines cohort study. Metabolism (2017) 67:62–71. doi: 10.1016/j.metabol.2016.11.002 28081779

[18] Guo W Qin P Li XN Wu J Lu J Zhu WF. Free triiodothyronine is associated with hepatic steatosis and liver stiffness in euthyroid Chinese adults with non-alcoholic fatty liver disease. Front Endocrinol (Lausanne) (2021) 12:711956. doi: 10.3389/fendo.2021.711956 34456869 PMC8387962

[19] Zhou J Tripathi M Ho JP Widjaja AA Shekeran SG Camat MD. Thyroid hormone decreases hepatic steatosis, inflammation, and fibrosis in a dietary mouse model of nonalcoholic steatohepatitis. Thyroid (2022) 32(6):725–38. doi: 10.1089/thy.2021.0621 35317606

[20] Kowalik MA Columbano A Perra A. Thyroid hormones, thyromimetics and their metabolites in the treatment of liver disease. Front Endocrinol (Lausanne) (2018) 9:382. doi: 10.3389/fendo.2018.00382 30042736 PMC6048875

[21] Hassan MM Kaseb A Li D Patt YZ Vauthey JN Thomas MB. Association between hypothyroidism and hepatocellular carcinoma: A case-control study in the united states. Hepatology (2009) 49(5):1563—1570. doi: 10.1002/hep.22793 19399911 PMC3715879

[22] Reddy A Dash C Leerapun A Mettler TA Stadheim LM Lazaridis KN. Hypothyroidism: A possible risk factor for liver cancer in patients with no known underlying cause of liver disease. Clin Gastroenterol Hepatol (2007) 5(1):118–23. doi: 10.1016/j.cgh.2006.07.011 17008133

[23] Shao YY Cheng AL Hsu CH. An underdiagnosed hypothyroidism and its clinical significance in patients with advanced hepatocellular carcinoma. Oncologist (2021) 26(5):422–6. doi: 10.1002/onco.13755 PMC8100545 33687750

[24] Liao CH Yeh CT Huang YH Wu SM Chi HC Tsai MM. Dickkopf 4 positively regulated by the thyroid hormone receptor suppresses cell invasion in human hepatoma cells. Hepatology (2012) 55(3):910–20. doi: 10.1002/hep.24740 21994129

[25] Frau C Loi R Petrelli A Perra A Menegon S Kowalik MA. Local hypothyroidism favors the progression of preneoplastic lesions to hepatocellular carcinoma in rats. Hepatology (2015) 61(1):249—259. doi: 10.1002/hep.27399 25156012

[26] Martínez-Iglesias O Olmeda D Alonso-Merino E Gómez-Rey S González-López AM Luengo E. The nuclear corepressor 1 and the thyroid hormone receptor β suppress breast tumor lymphangiogenesis. Oncotarget (2016) 7(48):78971–84. doi: 10.18632/oncotarget.12978 PMC5346691 27806339

[27] Ledda-Columbano GM Perra A Loi R Shinozuka H Columbano A. Cell proliferation induced by triiodothyronine in rat liver is associated with nodule regression and reduction of hepatocellular carcinomas. Cancer Res (2000) 60(3):603–9. 10676643

[28] Kowalik MA Puliga E Cabras L Sulas P Petrelli A Perra A. Thyroid hormone inhibits hepatocellular carcinoma progression *via* induction of differentiation and metabolic reprogramming. J Hepatol (2020) 72(6):1159–69. doi: 10.1016/j.jhep.2019.12.018 31954205

[29] Moreno M de Lange P Lombardi A Silvestri E Lanni A Goglia F. Metabolic effects of thyroid hormone derivatives. Thyroid (2008) 18(2):239–53. doi: 10.1089/thy.2007.0248 18279024

[30] Harrison SA Bashir MR Guy CD Zhou R Moylan CA Frias JP. Resmetirom (MGL-3196) for the treatment of non-alcoholic steatohepatitis: A multicentre, randomised, double-blind, placebo-controlled, phase 2 trial. Lancet (2019) 394(10213):2012–24. doi: 10.1016/S0140-6736(19)32517-6 31727409

[31] Kelly MJ Pietranico-Cole S Larigan JD Haynes NE Reynolds CH Scott N. Discovery of 2-[3,5-dichloro-4-(5-isopropyl-6-oxo-1,6-dihydropyridazin-3-yloxy)phenyl]-3,5-dioxo-2,3,4,5-tetrahydro[1,2,4]triazine-6-carbonitrile (MGL-3196), a highly selective thyroid hormone receptor β agonist in clinical trials for the treatment of dy. J Med Chem (2014) 57(10):3912–23. doi: 10.1021/jm4019299 24712661

[32] Runfola M Sestito S Bellusci L La Pietra V D’Amore VM Kowalik MA. Design, synthesis and biological evaluation of novel TRβ selective agonists sustained by ADME-toxicity analysis. Eur J Med Chem (2020) 188:112006. doi: 10.1016/j.ejmech.2019.112006 31931337

[33] Caddeo A Kowalik MA Serra M Runfola M Bacci A Rapposelli S. TG68, a novel thyroid hormone receptor-β agonist for the treatment of NAFLD. Int J Mol Sci (2021), 22(23):13105. doi: 10.3390/ijms222313105 34884910 PMC8657920

[34] Schroeder-Gloeckler JM Rahman SM Janssen RC Qiao L Shao J Roper M. CCAAT/enhancer-binding protein beta deletion reduces adiposity, hepatic steatosis, and diabetes in lepr(db/db) mice. J Biol Chem (2007) 282(21):15717–29. doi: 10.1074/jbc.M701329200 PMC4109269 17387171

[35] Abràmoff MD Magalhães PJ Ram SJ. Image processing with ImageJ. Biophotonics Int (2004) 11(7):36–42.

[36] Sato K Kitahara A Satoh K Ishikawa T Tatematsu M Ito N. The placental form of glutathione s-transferase as a new marker protein for preneoplasia in rat chemical hepatocarcinogenesis. Gan (1984) 75(3):199–202. 6724227

[37] Yamakawa H Kato TS Noh JY Yuasa S Kawamura A Fukuda K. Thyroid hormone plays an important role in cardiac function: From bench to bedside. Front Physiol (2021) 12:606931. doi: 10.3389/fphys.2021.606931 34733168 PMC8558494

[38] Dillmann W. Cardiac hypertrophy and thyroid hormone signaling. Heart Fail Rev (2010) 15(2):125–32. doi: 10.1007/s10741-008-9125-7 PMC2820695 19125327

[39] Sinha RA Singh BK Yen PM. Direct effects of thyroid hormones on hepatic lipid metabolism. Nat Rev Endocrinol (2018) 14(5):259–69. doi: 10.1038/nrendo.2018.10 PMC6013028 29472712

[40] Chi HC Chen SL Tsai CY Chuang WY Huang YH Tsai MM. Thyroid hormone suppresses hepatocarcinogenesis *via* DAPK2 and SQSTM1-dependent selective autophagy. Autophagy (2016) 12(12):2271–85. doi: 10.1080/15548627.2016.1230583 PMC5173272 27653365

[41] Chi HC Chen SL Lin SL Tsai CY Chuang WY Lin YH. Thyroid hormone protects hepatocytes from HBx-induced carcinogenesis by enhancing mitochondrial turnover. Oncogene (2017) 36(37):5274–84. doi: 10.1038/onc.2017.136 28504722

[42] Perra A Kowalik MA Pibiri M Ledda-Columbano GM Columbano A. Thyroid hormone receptor ligands induce regression of rat preneoplastic liver lesions causing their reversion to a differentiated phenotype. Hepatology (2009) 49(4):1287–96. doi: 10.1002/hep.22750 19115221

[43] Ito M Suzuki J Tsujioka S Sasaki M Gomori A Shirakura T. Longitudinal analysis of murine steatohepatitis model induced by chronic exposure to high-fat diet. Hepatol Res (2007) 37(1):50–7. doi: 10.1111/j.1872-034X.2007.00008.x 17300698

[44] Cao Z Sun X Icli B Wara AK Feinberg MW. Role of kruppel-like factors in leukocyte development, function, and disease. Blood (2010) 116(22):4404–14. doi: 10.1182/blood-2010-05-285353 PMC2996110 20616217

[45] Cvoro A Devito L Milton FA Noli L Zhang A Filippi C. A thyroid hormone receptor/KLF9 axis in human hepatocytes and pluripotent stem cells. Stem Cells (2015) 33(2):416–28. doi: 10.1002/stem.1875 PMC6317531 25330987

[46] Kowalik MA Guzzo G Morandi A Perra A Menegon S Masgras I. Metabolic reprogramming identifies the most aggressive lesions at early phases of hepatic carcinogenesis. Oncotarget (2016) 7(22):32375–93. doi: 10.18632/oncotarget.8632 PMC5078020 27070090

[47] Perra A Simbula G Simbula M Pibiri M Kowalik MA Sulas P. Thyroid hormone (T3) and TRbeta agonist GC-1 inhibit/reverse nonalcoholic fatty liver in rats. FASEB J (2008) 22(8):2981–9. doi: 10.1096/fj.08-108464 18434432

[48] Cable EE Finn PD Stebbins JW Hou J Ito BR van Poelje PD. Reduction of hepatic steatosis in rats and mice after treatment with a liver-targeted thyroid hormone receptor agonist. Hepatology (2009) 49(2):407–17. doi: 10.1002/hep.22572 19072834

[49] Kannt A Wohlfart P Madsen AN Veidal SS Feigh M Schmoll D. Activation of thyroid hormone receptor-β improved disease activity and metabolism independent of body weight in a mouse model of non-alcoholic steatohepatitis and fibrosis. Br J Pharmacol (2021) 178(12):2412–23. doi: 10.1111/bph.15427 33655500

[50] Harrison SA Bashir M Moussa SE McCarty K Pablo Frias J Taub R. Effects of resmetirom on noninvasive endpoints in a 36-week phase 2 active treatment extension study in patients with NASH. Hepatol Commun (2021) 5(4):573–88. doi: 10.1002/hep4.1657 PMC8034581 33860116

